# A Self-Regulation Intervention Conducted by Teachers in a Disadvantaged School Neighborhood: Implementers’ and Observers’ Perceptions of Its Impact on Elementary Students

**DOI:** 10.3390/children10111795

**Published:** 2023-11-08

**Authors:** Jennifer Cunha, Ana Guimarães, Juliana Martins, Pedro Rosário

**Affiliations:** Psychology Research Centre, School of Psychology, University of Minho, 4710-057 Braga, Portugal; jcunha@psi.uminho.pt (J.C.); id9998@alunos.uminho.pt (A.G.); juliana.oliveira.martins.psi@gmail.com (J.M.)

**Keywords:** disadvantaged school neighborhood, elementary teachers, fourth grade, knowledge transfer, narrative-based intervention, self-regulated learning

## Abstract

Self-regulated learning contributes to students’ academic success and their future as citizens. However, self-regulation skills are seldom or poorly promoted during instruction. To address this gap, the current article reports data on the implementation of an evidence-based intervention (i.e., a narrative-based intervention called “Yellow Trials and Tribulations”) in a disadvantaged school neighborhood. Prior studies showed positive results of this intervention in promoting elementary students’ self-regulation skills. Still, the data are mainly quantitative and limited to students’ reports or classroom observations made by researchers. Hence, the current study aimed to explore the implementers’ and observers’ perceptions of the impact of the intervention. Four elementary teachers implemented the intervention in their fourth-grade classes (N = 96 students). For each session, the implementers and observers completed a session sheet collaboratively, as well as individual final reports at the end of the intervention. The records were analyzed through a direct content analysis. The data indicated a perceived increase in knowledge and the use of self-regulation skills in the educational context and in daily life routines. Moreover, the data allowed for the identification of other positive gains of the intervention. The findings extended prior research while helping researchers to further understand the impact of the narrative-based intervention. The implications for research and educational practice are provided.

## 1. Introduction

Self-regulated learning (SRL) involves activating and sustaining thoughts, behaviors, and emotions toward self-set goals [1,2]. However, SRL is not always intentionally promoted in educational settings. The studies conducted by Spruce and Bol [3] and Dignath and Büttner [4] provide empirical evidence supporting this educational issue. In the first study, the authors collected data through self-reports, interviews, and classroom observations. The findings indicated that teachers (third to eighth grade) have a superficial knowledge of SRL processes, namely the planning and evaluation phases. In the second study, the authors analyzed elementary and secondary teachers’ data separately, which provided further insights. The data indicated that teachers, especially from elementary schools, “spent little time explicitly teaching SRL strategies”; concretely, the teachers dedicated less time addressing metacognitive strategies when compared to cognitive strategies [4] (p. 127). Moreover, the teachers were found teaching SRL strategies to their students using an implicit mode. Finally, the literature reports [3,5] that the limited SRL strategy instruction in class can be related to students’ poor self-regulatory skills.

The latter is a problem requiring attention from educators and school psychologists because extant research indicates that students with poor SRL skills tend to procrastinate, report lower motivation and academic outcomes, and have negative emotions toward school [6,7,8,9,10,11,12]. Furthermore, the literature indicates that even students with better academic results do not always use strategies to plan, monitor, and evaluate their learning [13,14]. This finding is even more critical for students from disadvantaged socio-economic contexts. For example, Evans and Rosenbaum [15] found that 9-year-old children from families of low socio-economic status, who often experience limited opportunities for learning enrichment (e.g., quality of interactions and extra activities such as music) and live in rural areas, have more difficulties regulating their behaviors as well as their emotions than their counterparts. Moreover, following a longitudinal design, these authors reported that this scenario of scarcity is likely to be reflected in poor school grades at 13 years old and is mediated by SR skills [15].

These findings highlight the importance of conducting direct interventions to promote students’ SRL skills at earlier stages [16,17] while involving teachers’ training [3,4]. However, implementing sound and empirically supported interventions into practice may take decades [18] and is often out of the school budget [19]. Considering these obstacles, funding empirically supported school interventions is valuable, especially in disadvantaged neighborhoods. In Portugal, a national foundation financed the implementation of evidence-based interventions in the community, facilitating knowledge transfer [20]. The current study was conducted in this context and aimed to explore the implementers’ and observers’ perceptions of the impact of the SRL intervention called “Yellow Trails and Tribulations” on the participating elementary classes from a disadvantaged school neighborhood. The analysis followed a qualitative approach centered on the implementers’ and observers’ records (i.e., session sheets and final reports).

### 1.1. SRL: Theoretical Framework

SRL is one of the major topics studied in the educational psychology field, presenting various theoretical frameworks [21]. Zimmerman’s model is one of the most cited and applied models in the classroom context. This theoretical model provides a relevant theoretical framework to the current study, and it will be briefly described hereafter.

The model presents the processes and subprocesses of SRL organized into three phases (i.e., forethought, performance or volitional control, and self-reflection), through which students set goals, monitor their progress, and reflect upon their performances [22]. This model has a cyclical nature; each phase is influenced by the one immediately preceding it and influences the following phase [22].

The first phase refers to the processes preceding the efforts to complete, for example, an academic task: task analysis and self-motivational beliefs. Task analysis involves setting proximal goals and planning strategies that are suited to the task at hand [23]. Moreover, Zimmerman [22] considered various self-motivational beliefs underpinning this phase: (a) self-efficacy (i.e., an individual’s beliefs of their ability to learn or perform effectively) [24], (b) outcome expectations (i.e., beliefs about the ultimate ends of performance) [24], (c) task interest/value (i.e., individuals’ perceptions of the importance of the task), and (d) goal orientation (i.e., focus on the learning process or the product such as grades). The forethought phase influences the performance phase, where students are expected to complete tasks that activate the self-control and self-observation processes [22]. Self-control processes involve techniques (i.e., self-instruction, imagery, attention focusing, and task strategies addressed in the first phase) to focus on the task and optimize efforts. Self-observation, through metacognitive monitoring and self-recording, guides students’ self-control processes [2,22]. The performance phase influences the self-reflection phase, which encompasses two main processes (i.e., self-judgment and self-reaction) as reactions to students’ outcomes. Self-judgment involves self-evaluating results using a criterion (e.g., self-progressing toward learning goals, others, or standards) and is related to two key forms of self-reaction: self-satisfaction and adaptive reactions. Students exhibiting an adaptive behavior are likely to adjust or change their behaviors to improve results; conversely, those displaying a defensive reaction may avoid making any changes [22]. Self-satisfaction and self-reactions will positively or negatively influence the next forethought phase depending on whether students feel positive emotions and adopt adaptive behaviors or feel negative emotions and adopt defensive behaviors after the outcomes of their actions [2].

Despite this brief description of the (sub)processes of Zimmerman’s model, it is possible to conclude that SRL not only implies motivational, cognitive, behavioral, and emotional processes but also metacognitive processes to achieve self-set learning goals [2,21]. Metacognition is thus a process that is closely related to SRL and has been extensively studied in the educational psychology field [25]. Flavell [26] defined metacognition as the knowledge concerning one’s cognitive processes and products, and it is generally understood as involving (i) self (e.g., competencies), task (e.g., difficulty), and strategy (e.g., concept map) knowledge; (ii) metacognitive experience (e.g., what students consciously think and feel during the process); (iii) goals (i.e., what the student wants to achieve); and (iv) actions or strategies toward the goals. According to this author, monitoring occurs during the interaction of those processes. Schraw’s work provides a more direct link to Zimmerman’s SRL model and a helpful application for educational practice. Accordingly, metacognition encompasses two main related components: knowledge and regulation [27,28]. The knowledge component includes three typologies: declarative (e.g., what students know about; for example, what a diagram is), procedural (e.g., how to apply a strategy; for example, learning how to make a diagram), and conditional (e.g., when and why to apply declarative and procedural knowledge). Declarative and procedural knowledge do not suffice. Students are expected to use conditional knowledge to help them adjust their behaviors and resources to the context (e.g., the available time and demanding task). Simply put, the second component of metacognition refers to the way students control their learning through three skills: planning (e.g., predicting and selecting suitable strategies), monitoring (e.g., on-task thinking and comprehension about how the task is being performed), and evaluating (i.e., analysis of the final product and assessing to what extent the task was carried out successfully) [27,28]. Strategic learners are likely to score high in both components [29].

The processes and subprocesses of SRL and metacognition are malleable, and they can be promoted by educators (e.g., parents and teachers) through the following sequence: direct instruction, modeling, guided practice, and feedback [1,28,30,31]. Moreover, several programs to promote SRL competencies (with some emphasizing the metacognitive processes) have been developed and empirically validated in recent decades. For example, extant reviews and meta-analyses contributed to mapping SRL programs and summarizing the characteristics that most likely contribute to their effectiveness in compulsory and higher education [16,32,33,34,35]. Due to the purposes of the current study, the following section will provide a brief overview of SRL programs in elementary school.

### 1.2. SRL Interventions for Elementary Students: What Seems to Work?

The meta-analysis conducted by Dignath, Büttner, and Langfeldt [33] is one of the most valuable works in SRL research at the elementary school level. This work compiles SRL training programs and their characteristics (e.g., the content of the intervention, theoretical background, and the implementer) that had the highest effects on elementary students’ overall performances, domain-specific performances, strategy uses, and motivational variables (whenever possible, recent references will be added to this overview).

Overall, there is a large number of SRL interventions, differing in the number and age of participants, domain specificity (e.g., math subject, reading, writing, and science) or non-domain specificity, intervention duration (e.g., two sessions, eight sessions, or ninety sessions), the implementer of the program (i.e., teacher or researcher), session content, format, and the instruments used to assess the effectiveness of the intervention [16,17,33,35,36,37]. Moreover, there are differences related to the theoretical backgrounds of the supporting interventions. The programs may be grounded on motivational [38], metacognitive [39], socio-constructivism [40], or socio-cognitive models [30,35,41], or even a combination of models [35,42].

In brief, Dignath, Büttner, and Langfeldt [33] found that the most effective interventions were those (i) grounded on socio-constructivism, which provided knowledge about the use of strategies and their benefits; (ii) designed to provide students with opportunities to learn problem-solving strategies and strategies for elaboration (cognitive strategies), planning (metacognitive strategies), and feedback (motivational strategies); and (iii) conducted by researchers (overall, recent meta-analyses conducted in subsequent school levels showed similar results [32,34,43]). Following this work, many interventions focusing on elementary students were developed and investigated [35]. Besides those, the narrative-based intervention (“Yellow Trails and Tribulations”) developed by Rosário et al. [44,45] has been implemented and assessed in the past 14 years [41,46,47,48,49].

### 1.3. The “Yellow Trails and Tribulations” Narrative-Based Intervention

The narrative called “Yellow Trials and Tribulations” [44] tells the story of the disappearance of the color Yellow from the rainbow and the adventures lived by the other colors while in search of their friend (see Table A1). Grounded on the social cognitive theory, the intervention using this narrative was designed to promote a set of SR strategies (e.g., setting goals; monitoring tasks; and self-evaluation) among children under the age of 10 without focusing on a specific domain or subject [50]. These strategies are presented by the narrative characters throughout 17 chapters, functioning as role models to children [51].

The intervention using this narrative was designed to be flexible without a rigid number of sessions. However, the sessions must follow the same sequence (see details in the Procedure subsection). After reading each chapter, following a metacognitive approach, children are encouraged to reflect upon and train each SR strategy [17,50]. For example, children are trained to develop declarative (e.g., what is a goal), procedural (e.g., how to set a goal), and conditional (e.g., when and why they should set a goal) knowledge of the SR processes and strategies in academic and non-academic domains [17,50]. The theoretical basis supporting the intervention program’s design is the PLEE model (meaning Planning, Execution, and Evaluation) based on Zimmerman’s SRL model [22], which was previously described. The PLEE model adds a recursive element to the previous model; the three phases of the cycle are set within each phase [17,50]. Specifically, the planning phase refers to the preparation for the execution phase. It involves thinking about the reasons to perform a task and setting strategies to attain pre-established self-goals. Planning requires thinking about what, when, where, and how to do something, and whether help is needed [50]. The execution phase requires putting the plan into practice and monitoring its process [50]. The last phase involves assessing whether the tasks progressed as planned and questioning the reasons for the positive and negative outcomes [50].

Throughout each chapter of the narrative, one or more phases of the PLEE model are addressed with different levels of detail. For instance, the importance of the planning phase is introduced in the second chapter, and it is then further explained in the following chapter by the “Smiling Eagle” character. This character explicitly defines the planning phase and provides an example of its application when hunting a rabbit. The execution and evaluation phases are then defined in the sixth chapter by the “General-Ant” character who also explains how the Ant Army follows the PLEE model [44]. In the subsequent chapters, the main characters use the PLEE model in different situations to overcome various challenges with a growing level of complexity (see Table A1). This provides several opportunities for children to reflect upon SR strategies—an educational approach termed the “onion approach” [50] (p. 150).

This narrative based-intervention has been implemented by researchers for fourth-graders (including students from minority or low socio-economic backgrounds) living in urban or rural areas [30,46,47,48,52,53,54]. The results showed positive and significant impacts of the intervention on (i) the participants’ self-reported SRL competencies and self-efficacy beliefs [46,48,52,53,54], (ii) the observational cognitive engagement components related to SRL such as asking for help and verbalizations related to the task planning [30], and (iii) attention control, which was assessed using a standardized test (impressively, at the end of the intervention, the experimental group scored at the level of 13/14 years old, while the control group scored at the level expected for their age group [54]). A particular highlight of the study conducted with students from low socio-economic backgrounds was that students presenting low and medium scores of SRL in the pre-test showed the highest gains in the post-test with high effect sizes [46].

More recently, the researchers conducted studies in which class teachers implemented the intervention after being trained by the research team for this purpose [49,55]. These studies showed positive results. At the end of the intervention, third and fourth graders reported higher uses of SRL compared to the pre-test [49]. Importantly, the effect size was d = 0.77, which is almost large. Tuero et al. [55] found long-term effects of this intervention in the follow-up (i.e., three months after) in terms of the students’ overall performance, specifically in the sciences and in the Spanish and English languages (although with small effect sizes).

The aforementioned studies used quantitative research methods to analyze the impact of the “Yellow Trails and Tribulations” intervention. However, some ad hoc qualitative data were collected in a post-research meeting aimed at sharing the preliminary results with the teachers of the participating students and collecting comments or suggestions that could help to enhance future intervention implementations [53]. In this session, the teachers shared their perceptions of the benefits of the intervention. For example, as Rosário et al. [53] reported, the teachers noticed that their students applied some lessons that were learned in other contexts (e.g., using PLEE in their daily activities, such as planning games on the playground). Despite being scarce, these data indicated the need to expand the knowledge on the impact of the aforementioned intervention through qualitative research.

### 1.4. The Present Study

Self-regulation skills are essential in school and lifelong learning [56] and help citizens succeed in various areas of life in the current competitive world [57,58,59]. However, several issues arise that prevent the development of SR skills in students in the school context: (i) teachers’ lack of knowledge of SRL processes and strategies, which is reflected in their insufficient explicit teaching [4,16]; (ii) the use of teacher-centered approaches rather than autonomy-supportive environments, primarily for students from disadvantaged backgrounds, because teachers seldom form high academic expectations for these students [37]; (iii) the massive gap between research knowledge and practice despite extant validated SRL interventions [16,18]; (iv) the fact that SRL interventions are often implemented by class teachers, which, based on prior meta-analyses, show lower effect sizes than those implemented by researchers [33]; (v) the fact that SRL interventions implemented by class teachers do not only require initial training, but it is also necessary to establish partnerships between teachers and researchers and monitor their practices [16,60]; and (vi) SRL interventions, despite being efficacious, are very costly (e.g., Self-Regulated Strategy Development [19]). These interventions are unaffordable for many schools, especially those with disadvantaged backgrounds.

Acknowledging these constraints, it is imperative to facilitate the implementation of evidence-based interventions in schools by supporting research–community partnerships. With this purpose, the Calouste Gulbenkian Foundation created the Gulbenkian Knowledge Academies (GKA). The GKA were designed to support the implementation, monitoring, and assessment of evidence-based interventions at a local level, aiming to promote children and youth socio-emotional skills [20]. The current paper refers to one funded evidence-based “Yellow Trails and Tribulations” intervention targeting elementary students to promote self-regulated learning [44,45].

As prior studies showed, this intervention enhanced elementary students’ SRL skills, including those from disadvantaged backgrounds [30,46]. However, the literature lacks research on the process of students’ change throughout their participation in the intervention. Hence, the current study aimed to explore the implementers’ and observers’ perceptions of the impact of the SRL intervention, “Yellow Trails and Tribulations”, throughout its implementation on the participating elementary classes from a disadvantaged school neighborhood. This study adds to the literature by providing information about the benefits of the aforementioned intervention that quantitative approaches cannot capture [61], for example, micro progresses [60].

The following research question guided the current study: what are the implementers’ and observers’ perceptions of the impact of the evidence-based intervention, “Yellow Trials and Tribulations”, on students?

## 2. Materials and Methods

### 2.1. Study Context

The current study refers to the implementation of the evidence-based intervention, “Yellow Trials and Tribulations”, in a school district in Portugal. The CGF funded this research work. The intervention setup followed three sources of information as follows: (i) school application form for GKA funding, (ii) educational needs assessment conducted by the research team, and (iii) data from the 2011 Census (although the project application occurred in 2018, the 2011 Portuguese Census was the report available to date). It is important to note that detailed information was omitted to avoid the risk of inadvertently revealing the participating schools.

The intervention was implemented in four 4th-grade and five 5th-grade classes of a school district where the educational offer ranges from preschool to high school (courses for adults are also available). In the GKA’s application form, two major problems were reported: a high number of students with one or two retentions and an increase in the number of students dropping out just after turning 18 years old (age of majority). The school principal applied for GKA funding for three years.

The participating schools are located in one of the largest regions of Portugal. Regarding the population characteristics, the average age is higher than the national average, and the Potential Sustainability Index (number of working-age individuals for each elderly) is significantly below the national average. Regarding education, the illiteracy rate is almost double the national rate, and the percentage of people aged 23 years or older holding a higher education degree is about 3 times lower than the national rate [62]. These data patterns are consistent with those reported in 2021 [63]. Individuals of this region are employed predominantly in activities of the secondary (manufacturing or industrial) and tertiary (services) sectors, with wages significantly below the national average [64].

The school principal characterized the schools as being located in a “dormitory town” where parents seldom engage in their children’s school lives. The principal perceived this as a problem related to students’ poor academic results and school dropout rates, which is consistent with the literature [65]. Another problem reported by the school principal was the parents’ and students’ low academic aspirations.

Given the aforementioned information regarding various indicators (e.g., social portray, education, employment, and salary), the research team concluded that the participating students were from a disadvantaged school neighborhood [66]. As mentioned, students from disadvantaged backgrounds often display fewer SR skills and lower academic achievements than their peers [15]. These data are consistent with the characterization of the students provided by the school principal.

### 2.2. Participants

During the 2018–2019 school year, the intervention was implemented in the 4th and 5th grades of the school district. The present study focuses only on 4th-grade students. Four classes were targeted for intervention, reaching 96 students (53.3% were female; six students did not report this information) with ages ranging from 8 to 10 years old (M = 9.27; SD = 0.52). More than 50% of the parents reported having a job not requiring specialization.

Eight female teachers participated in the study. Four acted as implementors of the intervention, and four acted as observers. The research team also trained the observers. The teachers’ teaching experiences ranged between 24 and 39 years (M = 28.25; SD = 8.26). One teacher had postgraduate training. Observers did not provide personal data.

### 2.3. Procedure

The current study was reviewed and approved by the Ethics Committee of the University of Minho. The guardians of the students who were enrolled in the intervention gave written informed consent for their child’s participation, following the Declaration of Helsinki. Teachers also signed informed consent forms regarding their participation in the study.

Prior to training, the research team and the school principal set a strategy to respond to the school’s needs during the three-year grant. In Year 1, all elementary school teachers (1st to 4th grade) and 5th-grade teachers would be enrolled in the initial training; however, only 4th- and 5th-grade teachers would implement the intervention. The teachers of the remaining school grades (from 1st to 3rd grade) would be observers. In the following two years, the teachers who had previously implemented the intervention during Year 1 would be observers, while 4th-grade teachers in Year 2 and then in Year 3 would implement the intervention. The goal of this organization was that by the end of the funding period, all elementary school teachers should be fully trained and able to autonomously conduct the intervention without the support of the research team. This study is focused on the implementation set at Year 1.

Between September and December 2018, 14 teachers from the 4th and 5th grades were enrolled in a 50 h training composed of a theoretical part (e.g., motivation theories and SRL models) followed by a hands-on component (e.g., preparation of a session and simulation of a session). The training was blended (i.e., 14 h of presential training, and the remaining hours occurred via an online platform) and delivered by the research team. After this formal training, teachers continued working on the intervention materials to better accommodate the contents, and they selected the chapters and goal(s) for each session. The final pool of chapters and session goals was agreed upon between the implementers based on the classes’ everyday needs.

**Figure 1:**
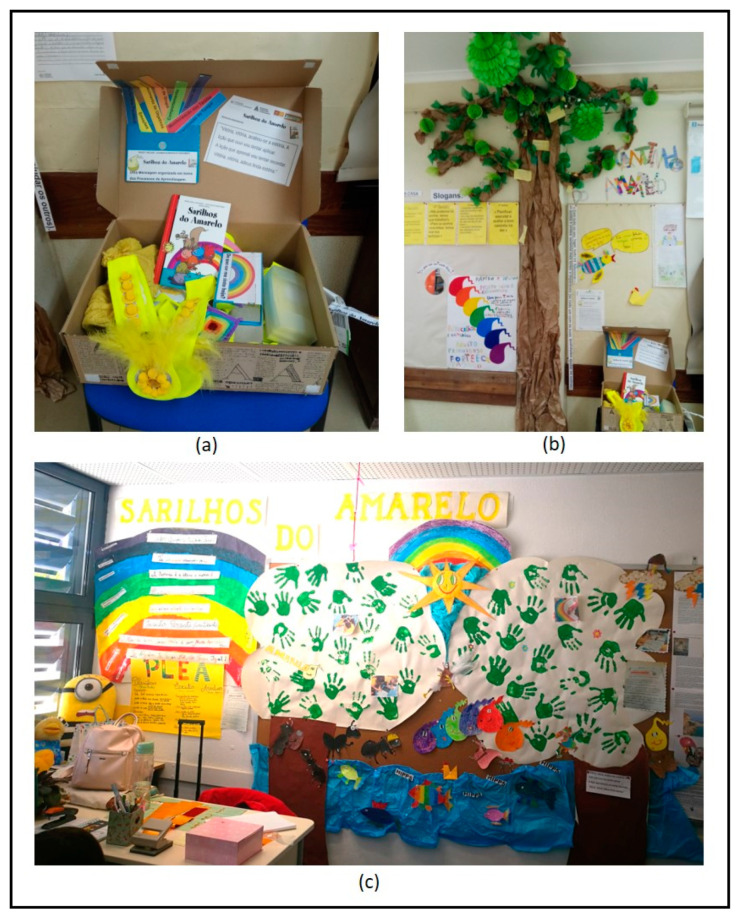
Examples of “Yellow Imaginary” settings: (**a**) box with the session materials; (**b**) “Yellow Corner”—Example 1; (**c**) “Yellow Corner”—Example 2.

In mid-March 2019, the 4th-grade teachers started the 10-week implementation (see Table A1). The intervention protocol indicated an implementation time of 60 min once a week. Ten 60 min sessions is the minimum time needed to implement the intervention, and prior research showed positive results in students from disadvantaged backgrounds [46]. The intervention was carried out in the classroom during instruction time. Sessions were structured according to six components [45,50]: (i) setting the scene (i.e., creating the “Yellow imaginary setting” (throughout the intervention, the students, with the implementer’s support, developed the “Yellow Corner”, where they created some scenarios of the story and exposed their works (see Figure 1)); for example, at the beginning of all sessions, the students and the teacher put on a yellow hat or necklace (see Figure 1)); (ii) reviewing the previous session and lessons learned; (iii) reading the chapter(s) of the “Yellow Trials and Tribulations” narrative [44]; (iv) exploring and discussing the chapter(s); (v) completing a practical and consolidation activity; and (vi) writing a take-home message (i.e., a sentence that emphasizes the content discussed). The reflection and discussion of the SR processes and strategies embedded in the chapters followed the three types of knowledge [17,67].

**Figure A1:**
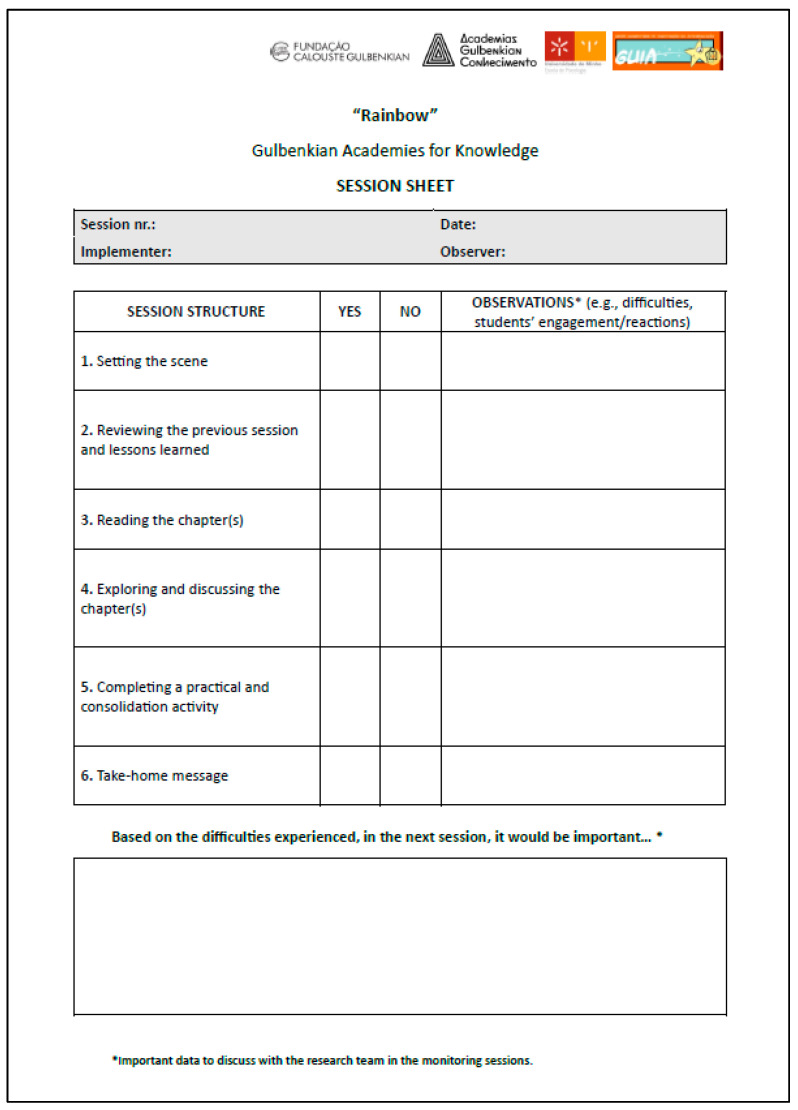
Session sheet.

The implementers and observers had to fill out a session sheet (see Figure A1) with multiple purposes as follows: (i) help implementers to self-monitor their practice, (ii) assess the adherence to the intervention protocol, (iii) provide support for implementers’ difficulties, and (iv) collect qualitative data to further understand the impact of the intervention on students. During the implementation of the intervention, the observers took notes to help fill out this session sheet, and at the end of each session, the implementers and observers filled it out collaboratively. Once a month, the research team, implementers, and observers met via videoconference.

### 2.4. Treatment Integrity

To assure the integrity of the intervention implementation, five procedures were adopted [68]: (i) intervention manual [45], (ii) teachers’ training, (iii) session protocol, (iv) session sheets, and (v) practice monitoring by the research team. The intervention manual was provided at the beginning of the teachers’ training. Teachers’ training involved indirect (instruction about the intervention) and direct (e.g., role-play of one session with researchers’ feedback) procedures [68]. The session protocol (i.e., major and specific goals, steps of the session, questions to ask at each step of the session, and practical activity) was adapted by the teachers with the support of the research team (e.g., from the intervention manual, teachers selected specific goals for each session and activities to conduct with students to meet their needs). Regarding the implementation of the intervention, at the end of each session, teachers and observers filled out the session sheet collaboratively (see description below). Afterward, the implementers emailed the session sheets to the research team. An indirect assessment of the intervention adherence was conducted by analyzing the session sheets [68]. For example, one issue identified in the session sheets was related to time management (i.e., overall implementers and observers reported spending 15 more minutes than indicated in the protocol). The research team analyzed the session sheets and discussed issues related to the implementation of the intervention with the teachers through videoconference every month. These online meetings allowed for questions to be answered, instigated reflection, addressed difficulties, and provided strategies to minimize or overcome them.

### 2.5. Data Collection

Data were collected through two sources of information: session sheets and final reports. The session sheets consisted of a monitoring checklist organized according to the session structure (see Figure A1). Alongside the checklist was a white space to take notes on any challenges that arose during the session implementation and on students’ participation. At the end of the document, implementers and observers were encouraged to indicate the aspects needing improvement for the following sessions. The researchers asked the implementers and observers not to write students’ names in the session sheets for ethical reasons. Instead, they used a code provided by the research team coordinator.

After the intervention, each implementer and observer wrote a report about the intervention implementation. The report included the following topics: (i) brief contextualization/description of the implementation, (ii) perceived impact of the project on the students, (iii) discussion of the results, (iv) limitations/difficulties experienced in the field, and (v) suggestions for future implementations. Due to their relevance to further understanding the students’ progress over the intervention, the current study analyzed in detail the second topic of the report (i.e., the perceived impact of the project on the students). The ethical considerations regarding students’ information were also applied to the final reports.

### 2.6. Data Analysis

The session sheets and reports were analyzed using a directed content analysis method [69]. QSR International’s NVivo10 was used to assist with all data analyses [70]. Following a deductive approach, a codebook (see Table 1) was developed based on the PLEE theoretical model [50] before data analysis. Firstly, researchers read the implementers’ and observers’ materials to obtain an overall idea of the data. Subsequently, a deductive approach was used to code the data and fit the data into the theoretical-driven categories (i.e., planning, execution, evaluation, and PLEE). Importantly, at least one aspect of each phase description provided in the codebook was mentioned to code data in the categories mentioned. Data could be related to students’ SR knowledge (e.g., defining what planning is or how students can monitor and providing a hypothetical example) and/or skills (e.g., students’ experiences of how they applied PLEE, and not just mentioning how to use PLEE in a given activity).

As the coding process progressed, researchers noticed that implementers and observers mentioned other students’ gains from participating in the intervention. For this reason, following an inductive approach, data that did not fit into the SRL theoretical categories were coded using implementers’/observers’ words (e.g., class participation, peer relationship quality, and enthusiasm). In the end, a major category (i.e., other gains) and subcategories (i.e., teamwork and peer relationships, student participation, and positive emotions) were created to summarize all codes (see Table 1). The researchers believed that using deductive and inductive approaches contributed to a better understanding of the benefits of the “Yellow Trails and Tribulations” intervention.

From a total of 40 session sheets, 33 with relevant information for the study were coded. Two researchers independently analyzed the data. A consensus was reached after discussing the discrepancies in the codification. For example, the researchers decided to uncode “study timetable” because they did not have enough information regarding who and how students carried out their study timetables. This decision was based on the information gathered from a videoconference monitoring session. This helped the research team learn that students first outlined the study timetables as dictated by their teachers, revealing external regulation, and not self-regulation. The inter-observer agreement was calculated to ensure the coding scheme’s precision. According to Landis and Koch, the kappa value was 0.97, indicating an “almost perfect” agreement [71].

## 3. Results

The current study analyzes the session sheets completed by the implementers and observers as well as their final reports on the implementation of the intervention and its benefits for the participating students. The results are organized according to each phase of the PLEE model (i.e., planning, execution, and evaluation) and then according to the PLEE model as a whole. Finally, other gains of the intervention are described to expand the knowledge about the impact of the intervention.

**Figure 2:**
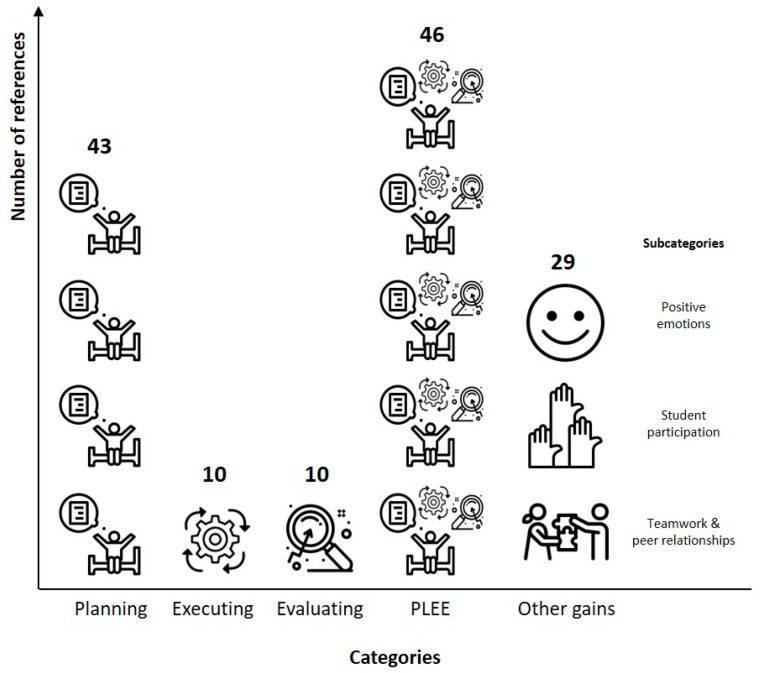
Graphical representation of findings.

Figure 2 provides a graphical representation of the overall findings, and Table 2 provides findings throughout the implementation of the intervention, from the first to the tenth session. As Figure 2 illustrates, the most mentioned categories were PLEE and planification, with a similar number of references, followed by other gains, and finally, executing and evaluating. Table 2 presents the perceived gains of the intervention in each session. The implementers and observers reported benefits since the first session.

Throughout the subsections presented below, exemplary quotes from the implementers’ and observers’ records were embedded to illustrate the findings. Frequently, the implementers and observers quoted the participating students, and some of them wrote the codes of the students. Considering this information, a matrix coding query crossing nodes (i.e., student code) by attributes (e.g., prior academic achievement) was conducted. Among the students mentioned in the session sheets, 26 were referenced once, and 10 were referenced at least twice. Twenty-three percent of the former and thirty percent of the latter showed a medium–low prior academic achievement.

### 3.1. Planning Phase

The planning phase was referenced in all session sheets except for the first one (see Table 2). From the second session onwards, the implementers/observers wrote that the students were able to define the planning phase, recognize its importance while performing tasks, and provide examples of its usage in different contexts. Examples of students’ quotes during a class discussion in the third session are as follows: “planning is to organize what has to be done—M told” (C4SS3) and “planning is thinking very carefully about what we want to do, how we are going to do it and how we will manage everything we need to solve a challenge” (C2SS3). The implementers/observers also registered examples of the application of planning to school tasks, such as solving math problems (e.g., reading the problem and extracting relevant information to understand what to do) and preparing for an assessment test, as the following quote illustrates: “to study I have to split the topics through the days [available before the test]—I told” (C2SS5). In the following sessions, the answers included more complex aspects such as setting goals (e.g., planning how to study to achieve their goal such as a grade of A), prioritizing tasks (e.g., deciding what is more important to do first), and developing schemes to organize ideas and study the contents for assessment tests.

The planning phase was also mentioned regarding non-school-related tasks (e.g., preparation for the elementary school graduation trip). One of the examples that was most mentioned was the preparation of a backpack for school or physical education classes. The implementers/observers provided students’ quotes to illustrate their notes: “When I go to the physical education class, firstly, I think about what I have to put in my backpack. I’m planning. Then I go get the necessary things and organize everything very well—F told” (C4SS6).

### 3.2. Execution Phase

The first reference to the execution phase was found in the sixth session sheet (see Table 2). The implementers/observers reported that the students understood the importance of monitoring and being focused when performing a task. One of the implementers/observers described how the students realized the importance of monitoring by executing a chicken-shaped origami. To complete this task successfully, monitoring was key to verifying, step by step, whether the origami was similar to the model. Without engaging in monitoring, the students risk making incorrect folds to the paper, which may translate to the final shape being different from the model. Monitoring also helped the students perceive when they needed help to perform a specific origami fold before making a mistake.

Another example was preparing a Mother’s Day gift (one of the art activities of the teachers’ lesson plan). The students organized the materials and followed the teachers’ instructions and the models provided while performing the task. According to the implementers’/observers’ notes, the students applied the planning and executing phases, as the following quote illustrates: “firstly, we have to decide what to do and organize the materials to do well our gift” (C1SS5). By the end of the intervention, the students also highlighted the importance of persistence when performing a task.

### 3.3. Evaluation Phase

The fifth session sheet referenced the evaluation phase for the first time (see Table 2). The implementers/observers reported that the students were able to define, identify, and apply the evaluation phase in various daily life situations. Specifically, the students seemed to understand that evaluating compares the performed task against the outlined plan.

Regarding school tasks, the implementers/observers registered the following observation: “when the teacher checks homework on the board, we can evaluate whether we did it correctly by comparing our answers and the resolution on the blackboard—D told” (C2SS6). The students seemed to understand that the evaluation phase helped them to improve their learning.

Moreover, the implementers/observers also registered other examples provided by the students, which were non-school-related tasks. For instance, the students mentioned household chores such as cleaning the bedroom or making the bed and the implications of not evaluating their behaviors: “Sometimes I don’t make my bed correctly. So, I sleep in un-fitted bedsheets, and I get my feet out. If I evaluated [the output] after making the bed, I could realize that I needed to do it again properly” (C4SS8). By providing this example, the implementer/observer registered that the students were able to establish relationships between their behaviors and consequences.

Finally, according to the implementers’/observers’ notes, the students recognized the importance of the evaluation phase and the cyclical nature of the PLEE model. One of the implementers/observers registered the following quote: “We don’t always get things right at the first time. We have to evaluate, see what we need to change and do it again until we get it right—G told” (C4SS8).

### 3.4. PLEE

References to the category “PLEE” were found in both the session sheets and final reports. In the sixth session sheet, the implementers/observers described how they carried out the application of PLEE (i.e., the implementers chose to do a dessert) with the students. According to the implementers’/observers’ notes, this activity helped the students understand the SRL model easily, and most of the students recalled the General-Ant character’s sayings, explaining the PLEE model.

At the beginning of the following session (when summarizing the prior session), the implementers/observers registered that the students were able to recall the PLEE acronym and enthusiastically shared examples of how they applied PLEE to school tasks (i.e., complying the lesson plan and solving a math problem) and how they and their families applied PLEE to other types of activities during the weekend (e.g., baking a cake, organizing a trip, preparing clothes for the next day, and playing video games). All implementers/observers provided detailed examples by quoting their students; for example:

“On the weekend, I planned a trip to the beach, and I was the one who helped my mother to plan. I made a list of what we would need and the games and activities we wanted to do. The picnic was planned, not forgetting the bag to put the rubbish in. We did everything we planned, and in the end, we concluded that we forgot nothing because we used PLEE—C told” (C2SS7).

Another note was regarding a student who shared in class that his mother started to praise him due to his newly acquired ability to pack the hockey trolley without forgetting anything. The student said he could carry this out because he used PLEE; his mother used to pack the hockey trolley and was amazed at this change.

Overall, the implementers and observers reported that the students understood how to apply PLEE to their daily lives, as well as its usefulness in doing things correctly and efficiently, as illustrated by the following quote: “if we follow PLEE, we don’t waste energy unnecessarily, nor do we waste time—M told” (C2SS6).

In the final reports, the implementers and observers mentioned that the opportunities for applying PLEE at school and at home provided an everyday organizational model and an effective problem-solving path that was likely to help explain students’ educational success. Even students who did not participate in class were involved in the program sessions and gave examples of how to apply the PLEE model in their activities (e.g., going camping for a weekend at a campsite with a friend, doing homework, and playing checkers with family) as well as its usefulness to avoid being late for school and pack the school materials needed.

### 3.5. Other Gains

According to the implementers’ and observers’ notes in their session sheets and final reports, the intervention led to other positive gains: teamwork and improved peer relationships, student participation, and positive emotions. Due to the importance of these perceived gains to further understand the benefits of the intervention, these are reported hereafter.

One of the first and most frequently perceived gains was teamwork and improved peer relationships. From the first session onwards, the participating students seemed to understand the importance of a group and working as a team: “after the debate that we organized, the students showed that they have internalized that if they are a united group, everything goes smoothly, and the result is much more positive for everyone” (C4SS1), and “the students highlighted the importance of listening everyone and working as a team” (C1SS5). By the end of the intervention, almost all implementers and observers reported that the participating students developed more positive and stronger relationships with their classmates, showing more respect and warmth. Some of the implementers and observers reported some quotes, such as “I was able to create strong bonds with everyone” (C1SS10), “Now I have more friends” (C1SS10), and “Before [the program] I just played with my best friend, now I have more friends” (C1SS10). Finally, one implementer/observer wrote that she and the students noticed that the conflicts between classmates decreased.

From the fourth session onwards, another positive gain that was frequently reported was student participation during session discussions and in-class and out-of-class activities. For example, one implementer/observer noted in the fourth session sheet that “the discussions and students’ reflections of the chapters are becoming more and more interesting. The reflections and discussions are deeper than those of the first sessions” (C1SS4). Other implementers and observers wrote that the students wanted to read the narrative and give their opinions, which had not happened before. By the end of the tenth session, one implementer/observer noted that “almost all students were more participative [than usual before the intervention]” (C1SS10). At the end of the intervention, students with low achievements appeared to be more confident and participated more during small-group and whole-class discussions.

Another implementer and observer registered that the students were more focused and showed more positive behaviors instead of disruptive behaviors in class. This change was reflected in their learning quality. The implementers’ and observers’ final reports were consistent with those findings as illustrated in the following quotes: “over time, there was a growing increase of all students’ participation in an orderly and constructive way” (C2TFR), and “it was also clear that students’ participation has become more active” (C1TFR). Although less mentioned, the implementers and observers noted that some students started doing their homework more often and studying more.

Regarding student participation, the implementers’ and observers’ final reports were consistent with the session sheet data. They mentioned that the engagement with the story and chapter discussion was evident as the sessions progressed. Overall, throughout the program, there was an increase in the number of students who (i) participated in the discussions, (ii) were more task-focused, (iii) behaved properly, (iv) and provided examples of the application of content learning to their school and daily lives.

Finally, by the tenth session and in the final report, one implementer/observer registered that the students revealed positive emotions such as enjoyment and enthusiasm for having improved their behaviors and learning quality. They provided illustrative quotes from two students showing low achievement before the intervention: “My teacher no longer calls my attention in class so much, my behavior improved, I read more, I give my opinion, I’m very happy!—said T” (C2SS10), and “I learned fantastic things! Fantastic things that can change the world!—said H enthusiastically” (C2SS10).

## 4. Discussion

The current study explored the perceived impact of the evidence-based “Yellow Trails and Tribulations” intervention conducted by class teachers in a disadvantaged elementary school neighborhood through a qualitative analysis of the implementers’ and observers’ session sheets and final reports. Prior studies majorly used quantitative methods to assess the impact of the intervention conducted by researchers [30,46,54] and then led by class teachers [49,55] for research purposes. Regardless of the implementer (i.e., researcher or trained teacher), those studies indicated that the participating students reported higher SRL skills at the end of the intervention than their counterparts [30,49,54], namely students from disadvantaged backgrounds [46]. However, the evidence gathered did not provide information regarding the progress of the participating students, especially in the context of research knowledge transfer to educational practice. The current qualitative study addresses this gap and is expected to contribute to the ecological validity of the evidence-based “Yellow Trails and Tribulations” intervention.

The data from the session sheets indicated that from the first session onwards, the intervention seemed to have contributed to the students’ understanding of the importance of teamwork. This finding may be related to the content of the first chapter of the narrative, where the narrator says that in the Never-Ending Forest, everyone helps each other, and everything perfectly works together. Specifically, in the first session, the implementers discussed the meaning of the following sentences: “Everyone is at everyone’s service. Together they help each other, together they do wonders” [44] (p. 9).

In the second chapter, Yellow disappeared, and the rainbow colors worked together to find their friend. To reach this major goal, in each chapter, the colors had to learn and use a set of SRL processes and strategies and served as models to the children [17,50]. Moreover, through the reading, discussion, and intentional hands-on activities (with opportunities to receive feedback), the participating students were trained on the three types of knowledge [17,67]. This SRL training, following a metacognitive and motivational approach, is one of the characteristics that contributes to the positive impact of this intervention [33,49]. Specifically, from the second session onwards, the participating students progressively learned what, how, and when to plan, execute, and evaluate their behaviors (see Table 2) by working on both the knowledge and regulation components of metacognition [28]. Interestingly, a high number of references from the implementers’ and observers’ records was found for the planning phase and PLEE (see Figure 2). In contrast, a much lower number of references was found for the executing and evaluating phases. This low number of references may be due to the executing and evaluating phases and the PLEE model being introduced sequentially (i.e., chapters 4–6). Afterward, the students often explained the executing and evaluating phases using the PLEE model, which may suggest that the students understood its cyclical nature [17,50].

Overall, the current study corroborates the prior quantitative results [46,49] and expands the prior qualitative data [41,53] by indicating the participating classes’ overall progress throughout the intervention. The quotes that were gathered from the session sheets illustrate that the implementers and observers perceived an increase in the overall classes’ SR knowledge (e.g., when students were able to define the planning phase) and skills (e.g., examples of how students applied what they learned) used in school and daily life. These data provided several examples of knowledge transfer that were not captured in the previous quantitative analyses. Explicit training in the SR processes and strategies following a metacognitive approach was essential for the participating students to learn how to apply SR to school and other contexts and domains (at least those familiar to students, such as making the bed and sports). According to the literature, these are requirements for occurring knowledge transfer [72,73,74].

Besides SRL skill improvement, the data indicated other benefits such as teamwork and peer relationships, student participation, and positive emotions. As previously mentioned, teamwork was the first benefit mentioned by the implementers and observers. The intervention seemed to have led to more positive, warm, and respectful relationships between classmates and the emergence of new peer friendships. This new finding of the intervention merits further research, given that the literature indicates that positive peer relationships are related to engagement (e.g., behavioral) and academic achievement [75,76,77,78,79,80,81]. This is particularly important, as engagement is a prerequisite of students learning [82,83] and is one of the strongest predictors of academic achievement [84] and lifelong success [85].

Moreover, student participation during sessions and class discussions but also in out-of-class activities was identified. Mainly, the records mentioned students’ participation in session discussions, even from those who usually do not participate. This finding may be due to the structure of the narrative and sessions [86]. Throughout the narrative, all of the rainbow colors (i.e., characters) share their views and fears at different points of their journey. Therefore, the rainbow colors may have functioned as models [51] with which students identified and with whom they learned how to express themselves and participate in activities at their own pace [17,50,87]. The narrative, which was purposefully written without directly relating to students’ school experiences and failures, as well as the hands-on nature of the activities completed in the sessions, may have played significant roles in creating a friendly environment [88,89,90], which may have encouraged the students’ participation in the sessions [86]. Additionally, the reflection and discussion of the chapter after reading may have also instigated student participation [91]. Students’ participation during discussions fosters the learning of SR skills, which may help them improve both their in-class and out-of-class participation [92]. For example, Rosário et al. [17] found that the “Yellow Trails and Tribulations” intervention helped to improve the engagement (e.g., participation in class) of students from ethnic backgrounds. Considering those findings, researchers may expand the study to analyze the impact of the intervention on students’ classroom behavioral engagement and its relationships with out-of-class activities such as studying for tests and homework.

Although it was only mentioned by one implementer/observer, the positive emotions identified are also relevant, given their importance to students’ behavioral engagements and academic achievement [81]. As the findings illustrated, the students were happy and proud of their accomplishments. They understood the usefulness of what they learned, which is a motivational factor to reinforce their behaviors [93]. According to the literature [81,82,93], students’ emotions and affective reactions to class activities represent their interest and engagement. Students tend to be more emotionally engaged and show happiness and enthusiasm when performing meaningful and enjoyable learning activities [94,95]. Therefore, the positive emotions stemming from classwork should be acknowledged as a promising result, despite being mentioned by only one implementer, due to their influence on students’ behavior and, ultimately, on high-quality learning [93].

Altogether, the current study provides several indicators of the ecological validity of the intervention. It provides valuable data on the benefits of the intervention throughout its implementation, not only for SR but also for the engagement of classes from disadvantaged school neighborhoods. For example, the implementers and observers reported that even students who did not participate in the class wanted to share their opinions and improved their SR skills. Additionally, as the matrix coding query crossing nodes by attributes indicated, 23–30% of the students mentioned in the records showed medium–low achievement. These data support the literature, stressing that even students with more difficulties can improve [46]. The “Yellow Trails and Tribulations” narrative teaches students that “Those who don’t give up will succeed”.

### 4.1. Limitations and Future Research

The current research provided information on the progress of the participating classes’ SR processes and strategies throughout and at the end of the “Yellow Trails and Tribulations” intervention. The implementers and observers provided examples of the students’ gains in the session sheets and final reports and illustrated them with several students’ quotes. Notwithstanding the contribution of the current data, the findings are limited to the implementers’ and observers’ notes on the session sheets and final reports, which reflect their perceptions of the students’ overall change. Moreover, the implementers’ and observers’ notes and reports were not balanced, as they varied in the number and detail of the examples or quotes presented. For example, two of the four dyads of implementers and observers were more detailed in their examples and quotes than their counterparts. Future research may overcome these limitations by collecting data with students using, for example, diaries. This method of using microanalytic protocols would help to capture changes in knowledge and the use of overt and covert SRL strategies [96] for each student over time. Prior research showed that strategic and maximal learners reported high levels of SRL knowledge and application [29]. Moreover, researchers could triangulate data from implementers and students to gather deeper information on the benefits of the intervention. In addition, researchers could transform qualitative data into quantitative data and analyze, for example, the time needed to capture significant changes [49,67]. This mixed-method study would help to overcome the limitations of solely analyzing quantitative or qualitative data [61,97].

Another limitation of the current study is the absence of a follow-up. Using the methodology that was previously mentioned, it would be helpful to collect data three months after the intervention and explore whether the changes were maintained over time [46,49] or to analyze the students’ abilities to recall the contents that were learned in the intervention to solve complex problems in new situations (i.e., backward-reaching transfer [98]). This is particularly important in school transitions, when students face new educational challenges [90,99,100].

### 4.2. Implications for Practice

The challenges of transferring evidence-based interventions to the field, in this case, the school, is well documented in the literature [18,61]. In the case of SRL interventions, several obstacles were reported previously (e.g., teachers’ lack of knowledge of SRL processes and strategies, e.g., [4,16]; SRL interventions implemented by class teachers are less effective than those implemented by researchers, e.g., [33]; SRL interventions implemented by class teachers require more than just initial training, e.g., [16,60]; SRL interventions, despite being efficacious, are very costly, e.g., [19]. The current study is an example of how it is possible to overcome these difficulties.

Several aspects must be considered to implement SRL evidence-based interventions with observable benefits for the participating students. Firstly, there is a need to establish partnerships between researchers and the school (e.g., the school principal and the teachers who will implement the intervention). This partnership is essential to conduct a comprehensive needs assessment. In the case of the current work, the needs assessment helped to determine the logic of the work developed for the three years of funding. This planning is imperative to guarantee the sustainability of the intervention implementation and its benefits over time. School principals should consider this aspect from the beginning of any school-based intervention. Otherwise, all investments (e.g., teachers’ training and support; intervention materials) may be limited [101].

Afterward, researchers are expected to train the teachers to implement the intervention. The training is an important yet an insufficient requirement for effective interventions. There is a need to monitor their practice and provide ongoing support [16,60]. The use of session sheets is a useful tool to check the adherence to the intervention protocol and, most importantly, to note any difficulties to discuss with the research team and receive the support needed. The implementers must understand that the research team aims to provide support, and not to control their practice. In the case of the current study, the teachers initially perceived the session sheets as a subtle form of control. This is important to clarify because, as the literature warns, the perception of control negatively impacts teachers’ motivations and practices (e.g., [102]); on the other hand, the perception of support positively predicts not only motivation but also well-being [103].

As detailed in the Methods section, the intervention process requires several hours of training, monitoring, and support. This was possible thanks to the funding provided. Schools that are in need of particular interventions may find it useful to search for financing with national foundations or city halls [41] to be able to afford the implementation of evidence-based interventions that require costs with training, practice monitoring, and support, as well as materials for implementers (e.g., session guides) and students (e.g., narrative).

## Figures and Tables

**Table 1:** Codebook.

Category		Description
Planning		Preparing something. Process of “thinking before doing something”, which involves defining what, when, where, how, and with whom [44,45]. This phase implies setting a plan, goals, and strategies [50].
Executing		Doing something as planned before (e.g., using the materials and strategies selected, put the plan into practice in the time defined [50]). The process of “thinking while doing something” involves monitoring to check whether everything goes as planned [44,45]. This phase implies persistence and attention focusing.
Evaluating		Examining something finished. Process of “thinking after doing something”, which involves assessing whether something was accomplished as planned (e.g., completely as planned or whether there were delays [44,45]). This phase implies considering the reasons for a given result, positive or negative [50].
PLEE		Cycle of three phases: planning, executing, and evaluating [50]. Its application involves mentioning at least one aspect of each phase of the PLEE model.
Other gains	Teamwork and improved peer relationships	Recognizing the importance of working together collaboratively; having more friends; decrease peer conflicts; respect and warmth between classmates.
	Student participation	Frequency and quality of student participation during sessions and class discussions; positive classroom behaviors.
	Positive emotions	Feelings of joy, happiness, pride, and enthusiasm regarding school, school tasks, intervention, and performance.

**Table 2:** Findings throughout the implementation of the intervention according to class.

Class (Code)	Session Sheets										Final Report
	1	2	3	4	5	6	7	8	9	10	
1	---	Planification	Planification	Planification Other gains	Planification Evaluation Other gains	Planification Execution PLEE	PLEE	Planification Execution Evaluation PLEE Other gains	Planification Evaluation PLEE	PLEE Other gains	PLEE Other gains
2	---	Other gains	Planification	---	Planification	Planification Evaluation PLEE	PLEE	Planification Evaluation PLEE	Planification PLEE	Planification PLEE	PLEE Other gains
3	---	---	Planification	---	---	Planification	PLEE	PLEE	PLEE	Planification	---
4	Other gains	Other gains	Planification	Planification	Planification	Planification	PLEE	Planification Execution Evaluation PLEE	Planification Execution PLEE	Planification Execution PLEE	PLEE Other gains

Note: --- means the absence of findings.

## Data Availability

Data are contained within the article.

## Appendix A

**Table A1:** Ten-session “Yellow Trials and Tribulations” intervention: content of the narrative chapters and session goals selected by the implementers.

Session	Chapter(s)	Brief Descriptions of the Chapter(s) of the “Yellow Trials and Tribulations” Narrative [44]	Major Goal(s)
1	1	Introduction of the story: Story setting (e.g., Never-Ending Forest and the living beings that live there, where everyone helps each other and everything works together perfectly), and psychological description of the main characters (i.e., colors of the rainbow).	− Reflecting on the importance of peer and group work.
2	2	Presentation of the problem: The color Red notices that Yellow disappeared from the rainbow. The colors become worried and disorganized without knowing what to do. The Lizard Stone used to say, “There is a place for everything, and everything must be in its place” (p. 12). The color Red suggests searching for their friend Yellow. The color Green suggests that they could ask for help from the Hiccup River because he could know what to do.	− Identifying self-regulation strategies: organization and help-seeking. − Reflecting on the importance of organization and help-seeking in problem-solving.
3	3	The Hiccup River advises the colors not to give up and provides a clue: they should plan well what to do. The colors do not know what planning means. The Smiling Eagle appears and explains the meaning of planning and provides an example of its application when hunting a rabbit.	− Identifying the first phase of the self-regulatory process: planning.
4	4	The Teacher-Bird teaches a group of little birds how to fly, telling them that “learning more and better depends on what each one does” (p. 20) and “no one learns how to fly with the wings closed” (p. 21). The Teacher-Bird tells the story of the school of deer. One student deer does not want to jump or run and ends up being too heavy to jump and run in the woods. One day, he hurts his leg while competing with a grasshopper.	− Reflecting on the importance of persistence. − Reflecting on the importance of anticipating the consequences of behaviors.
5	5	The color Orange finds a message from Yellow (i.e., a chicken-shaped paper). The color Violet says they need to make a plan, and Green recalls that they need to prepare and think well about what to do before leaving, as the Smiling Eagle taught them. The colors start planning, and his friend, Squirrel, says, “To reach the top of a tree, you have to start climbing, but climbing one branch at a time” (p. 25). After a long journey with many challenges, the colors stop to rest.	− Identifying self-regulation strategies: goal setting. − Reflecting on the importance of goal division (i.e., divide a goal into small steps).
6	6 and 7	When the colors are resting, the attentive color, Violet, notices an army of ants passing by, and calls his friends (Chapter 6). The color Red asks the General-Ant whether someone saw Yellow. The General-Ant explains how the Ant Army follows the PLEE model and monitors their work. The colors recall the Teacher-Bird’s lessons. The colors find a quicksand swamp—an excellent opportunity to apply what they have learned from the Smiling Eagle and the General-Ant (Chapter 7). The colors set a strategy to achieve their goal (i.e., reach the other side of the swamp) and work together. The colors monitor their strategy step by step. The color Red uses a new strategy to overcome an obstacle, and the other colors help.	− Defining the three phases of the PLEE model (i.e., Planning, Execution, and Evaluation). − Reflecting on the importance of monitoring.
7	9 and 10	The color Blue finds the second message from Yellow—another chicken-shaped paper (Chapter 9). The colors continue their journey, walking many leagues, and when they stop to rest, they notice the preparation of the picnic of problems. At that picnic, there is a contest for the “Emperor of Problems”, and the main candidates are lies, laziness, pout, disobedience, and fear (Part I). The candidates present themselves and the arguments to be the “Emperor of Problems”. The contest for the “Emperor of Problems” takes place (Part II, Chapter 10). The color Violet finds a new message from Yellow (i.e., a chicken-shaped paper). The color Anil, who always pays attention to details, notices that the chicken-shaped paper is smaller than the previous one. The Smiling Eagle asks the colors how their PLEE is going and advises them to be careful of the danger in their way.	− Reflecting on the importance of analyzing feelings and behaviors. − Reflecting on the importance of anticipating consequences of behaviors over time (short-, medium-, and long-term).
8	13 and 14	The colors find the Pirate-Tree, which says that a message from Yellow is hidden in its branches (Chapter 13). However, the Pirate-Tree only shows the message if the colors unravel three riddles. The colors set various strategies and work together. The colors are successful in unraveling all of the riddles, and the Pirate-Tree becomes very angry because of that. The colors ask the Pirate-Tree to give them the message from Yellow (Chapter 14). The Pirate-Tree reveals that the message is hidden in a hollow trunk. The colors wonder why the tree lied and assess the situation to decide what to do. After a first inspection of the trunk, they notice that a large spider guards it. The colors use PLEE again (setting different strategies) to overcome the new challenge. The colors obtain the message from Yellow, which is smaller than the last one, and try to unravel the reason.	− Analyzing the steps of problem-solving. − Training the usage of PLEE in problem-solving. − Applying the steps of problem-solving in specific situations.
9	15 and 16	The colors sleep during the night, but their friend, Squirrel, wakes up the color Blue because he hears a groan—it could be Yellow asking for help (Chapter 15). The color Blue and Squirrel decide to leave to find Yellow without telling someone. Despite difficulties (e.g., dark fears), the two friends find a little lark that belongs to the Royal Choir of the Birds. The Lark is injured, and the two friends try to help. The Lark is very talkative, telling how she is so distractive and saying that singing in the choir is very difficult and the Bird-Maestro is so demanding. The two friends ask the Lark to let them think about what to do. It dawns, and the colors notice that the color Blue and the Squirrel are not there (Chapter 16). The colors become worried and disorganized. The color Orange, staying calm, tells the story of Hansel and Gretel. The color Green understands the point that Orange makes and proposes a strategy: instead of bread, they could use torches. The colors find their two friends and the Lark easily. The colors become angry, and their friends ask for forgiveness for their thoughtless act. The most important thing is to recognize when we make a mistake, apologize, and try not to do it again.	− Reflecting on the roles of mistakes in current and future behaviors. − Reflecting on the importance of taking responsibility for one’s own mistakes. − Rehearsing alternative behaviors in future events.
10	17	The Smiling Eagle wakes the group very early in the morning and asks how their goal is going. The colors say that they have not found Yellow yet. The Smiling Eagle asks whether they found the four messages from Yellow, and the colors wonder how the Smiling Eagle knows that. The Smiling Eagle admits that it has the last message, and that Yellow was hidden near the chicken camp, but Yellow disappeared again. The color Violet tells everyone to be calm and to think using PLEE, and the colors set a new plan with other strategies until they find Yellow (note: location not revealed purposefully). The colors celebrate. The rainbow is complete, and everything returns to the way it was in the Never-Ending Forest.	− Training and consolidating learning about self-regulatory processes. − Transferring learning about self-regulatory processes to other domains. − Celebrate achievements.

Notes: As planned, chapters 8, 11, and 12 were not read in the intervention sessions due to time constraints. Students read those chapters at home. Chapter 8: Exhausted, the colors lie down and watch the stars. The color Orange tells the story of Cassiopeia and explains how Perseus used PLEE to cut Medusa’s head. Chapter 11: The colors are worried about the warning of the Smiling Eagle, but they keep monitoring their steps. They stop to rest and dramatize the story of the Three Little Pigs (Part I). Chapter 12: Dramatization of the Three Little Pigs (Part II). The Smiling Eagle thanks them for the dramatization and says the colors applied PLEE very well in this activity. Some colors do not notice the application of PLEE, while others explain it.
